# Enhanced recovery after surgery (ERAS) program for elderly patients with short-level lumbar fusion

**DOI:** 10.1186/s13018-020-01814-3

**Published:** 2020-08-06

**Authors:** Peng Wang, Qiang Wang, Chao Kong, Ze Teng, Zhongen Li, Sitao Zhang, Wenzhi Sun, Mingli Feng, Shibao Lu

**Affiliations:** 1 Department of Orthopedics, Xuanwu Hospital of Capital Medical University, 45 Changchun Street, Xicheng, Beijing, 100053 People’s Republic of China; 2 National Clinical Research Center for Geriatric Diseases, Beijing, 100053 People’s Republic of China; 3 Department of Radiology, Cancer Hospital Chinese Academy of Medical Sciences, Beijing, 100021 People’s Republic of China

**Keywords:** Enhanced recovery after surgery, Elderly, Lumbar fusion surgery

## Abstract

**Background:**

Degenerative disorders of the lumbar spine decrease the mobility and quality of life of elderly patients. Lumbar fusion surgery is the primary method of treating degenerative lumbar spine disorders; however, the surgical stress response associated with major surgery has been linked to pathophysiological changes in the elderly, resulting in undesirable postoperative morbidity, complications, pain, fatigue, and extended convalescence. In the present study, we aimed to determine whether enhanced recovery after surgery significantly improved satisfaction and outcomes in elderly patients (> 65 years old) with short-level lumbar fusion.

**Methods:**

The study enrolled lumbar disc herniation or lumbar spinal stenosis patients if they were over the age of 65 years old underwent lumbar fusion at one or two levels. Data including demographic, comorbidity, and surgical information were collected from electronic medical records. Enhanced recovery after surgery interventions was categorized as preoperative, intraoperative, and postoperative. We also evaluated primary outcome, surgical complication, length of stay, postoperative pain scores, and 30-day readmission rates.

**Results:**

A total of 192 patients were included, 96 in the enhanced recovery after surgery group and 96 case-matched patients in the non- enhanced recovery after surgery group. There were no statistically significant intergroup differences in regards to demographics, comorbidities, American Society of Anaesthesiologists grade, or the number of fusion levels. There were also no differences between mean surgery time of intraoperative blood loss between the enhanced recovery after surgery and non- enhanced recovery after surgery groups. In addition, the mean preoperative Japanese Orthopaedic Association score, visual analog score for the back and legs, and Oswestry Disability Index score were not significantly different between the two groups. Overall, enhanced recovery after surgery pathway compliance was 92.1%. There were no significant differences in the number of complications or the mortality rates between the enhanced recovery after surgery and non-enhanced recovery after surgery groups. Furthermore, the mean postoperative Japanese Orthopaedic Association score, Visual analog score for the back and legs, Oswestry Disability Index score, and readmission rates score revealed no significant differences between the groups at 30-day follow-up point. However, we observed a statistically significant decrease in length of stay in the enhanced recovery after surgery group (12.30 ± 3.03 of enhanced recovery after surgery group versus 15.50 ± 1.88 in non- enhanced recovery after surgery group, *p* = 0). Multivariable linear regression showed that comorbidities (p = 0.023) and implementation of enhanced recovery after surgery program (*p* = 0.002) were correlated with prolonged length of stay. Multivariable logistic regression showed that no characteristics were associated with complications.

**Conclusions:**

This report describes the first enhanced recovery after surgery protocol used in elderly patients after short-level lumbar fusion surgery. Our enhanced recovery after surgery program is safe and could help decrease length of stay in elderly patients with short-level lumbar fusion.

## Background

With the improvement of people’s living standards and medical standards, the life expectancy and the number of elderly persons in the China continues to increase. The aging population has its own special characteristics, which are gaining increased research attention [1]. Generally speaking, elderly people over 75 years of age will experience reduced vital capacity, dysfunction of ventilation/blood flow ratio, osteoporosis, muscle atrophy, decreased muscle strength, decreased central regulatory function, reduced motor coordination, reduced strain control, and other pathophysiological changes, and the prevalence of degenerative diseases of the lumbar spine also increases [2].

Degenerative disorders of the lumbar spine may cause significant neural compression, increased pain, and a decrease in the mobility and quality of life of elderly patients. Furthermore, to help maintain their independence, more elderly patients who have failed conservative treatment of their lumbar spinal disorder, are looking toward a surgical solution. It is increasingly important to identify interventions that are effective at improving the quality of life of elderly patients with spinal disorders [3].

Lumbar fusion surgery is the main way for treating degenerative lumbar spine disorders; the surgical stress response associated with major surgery describes fundamental metabolic changes that lead to increased catabolism, immunosuppression, free radical production, and hypercoagulable states [4]. These physiologic alterations have been linked to changes in organ function resulting in undesirable postoperative morbidity, complications, pain, fatigue, and extended convalescence [5].

The benefits of enhanced recovery after surgery (ERAS) include reduction in the surgical stress response to minimize postoperative complications and after major surgery without increase in readmission rates, and increased patient satisfaction [4]. These decrease the surgical stress responses and are of particular importance for the vulnerable patient with comorbidities, who are often also frail and elderly [6]. Although ERAS has been shown to be effective for patients in general, few studies have addressed the effectiveness of ERAS in elderly patients with spinal disorders [7].

There is no single surgical method applicable to all lumbar degenerative diseases. The elderly suffer from recurrence after aperture surgery due to the decreased elasticity of the intervertebral disk; lumbar fusion surgery has its own advantages in the treatment of degenerative spinal diseases in the elderly. The aim of this study was to determine whether ERAS could significantly improve care in the perioperative period and decrease perioperative complications, in-hospital length of stay (LOS), and 30-day readmission rates in elderly patients (> 65 years old) with short-level lumbar fusion.

## Materials and methods

### Inclusion criteria and patient selection

This is a retrospective cohort study of prospectively collected data. The study protocol was approved by the Ethics Committee for Human Subjects of the Xuanwu Hospital of Capital Medical University (permit data 2018.4.3; no. 2018086). The study enrolled lumbar disk herniation or lumbar spinal stenosis patients if they were over the age of 65 years old underwent lumbar fusion at one or two levels from January 2018 to December 2019 (non-ERAS group), and between January to December 2019 (ERAS group). Both groups were cared for by the same surgical team. A retrospective non-ERAS group in which patients were treated under traditional perioperative protocols was case-matched to ERAS group. Diagnosis of degenerative disorders of the lumbar spine was performed by two spinal orthopedic specialists based on clinical symptoms and MRI images of the lumbar spine, which were used to identify the responsibility segments. Surgery was indicated when patients with typical symptoms of spinal stenosis did not respond to conservative treatments. Individuals who had infection disease, trauma, cauda equina injury, and neoplasm were excluded in this study, as well as those planned for a revision of a previous fusion.

Demographic data include age, gender, and body mass index (BMI). Comorbidities included hypertension, heart disease, diabetes, osteoporosis, stomach problem, bowel or intestinal problem, and psychological symptoms. Other interest included American Society of Anesthesiologists (ASA) physical status score, preoperative Japanese Orthopaedic Association Score (JOA), Oswestry Disability Index (ODI), and visual analogue score (VAS) for back and legs score. Operative records were reviewed to the number of fusion levels, operative time, and intraoperative blood loss. The primary outcome data that were analyzed included complication, length of stay, postoperative pain scores, and 30-day readmission rates. All data were collected from the electronic medical record.

### ERAS interventions

ERAS program was proposed and planned in 2017. The core group consisted of anesthesiologists, spine surgeons, nutritionists, physical therapists, physicians, geriatricians, and nurses. After literature review and experience discussion, a reasonable ERAS program was obtained. With the approval of the ethical committee for human subjects of the Xuanwu Hospital of Capital Medical University (Beijing, China), we began to implement the ERAS program in September 2018. Our ERAS interventions was divided into preoperative, intraoperative, and postoperative, included administration of the following: (1) patient education and counseling, (2) preoperative fasting, (3) antibiosis before surgery, (4) standard anesthetic protocol, (5) multimodal analgesia, (6) early feeding after surgery, (7) gastrointestinal management, (8) early mobilization medical, (9) early removal of bladder catheter, and (10) antithrombotic prophylaxis. The details of ERAS for pathway are presented in Table 1.

**Table 1:** Patient demographics

Patient demographics	ERAS	Non-ERAS	*p*
Sample size	95	95	
Age (years)	72.39 ± 6.12	70.81 ± 6.27	0.12
Male/female	45/50	40/55	0.47
Body mass index	25.67 ± 3.32	25.73 ± 4.00	0.93
Smoker	3	5	0.25
**Comorbidities**			
Hypertension	68	74	0.32
Heart disease	26	24	0.74
Chronic lung disease	0	2	0.16
Diabetes	24	31	0.26
Osteoporosis	16	15	0.84
Gastrointestinal	8	5	0.39
Psychological symptoms	3	2	0.65
Preoperative JOA	8.30 ± 2.11	8,12 ± 1.90	0.59
Preoperative ODI, %	60.89 ± 11.88	58.88 ± 8.26	0.55
Preoperative VAS (back)	7.15 ± 0.72	7.01 ± 0.70	0.25
Preoperative VAS (leg)	7.06 ± 0.63	7.09 ± 0.59	0.56
**ASA grade**			
I	13	10	
II	68	73	
III	14	12	
IV	0	0	
**No. of levels fusion**			
1	44	40	0.56
2	51	55	0.56
Operative time	186.78 ± 57.38	198.72 ± 69.48	0.58
Intraoperative blood loss	283.38 ± 195.44	311.51 ± 219.44	0.42

### Statistical analysis

All statistical analyses were performed using the SPSS software version 17.0 (SPSS, Inc., Chicago, IL, USA). Patient demographics, comorbidities data, markers of baseline health, and clinical outcomes were compared between ERAS group and non-ERAS group using Student’s test and *χ*^2^ test. Multivariable linear regression analysis was used to assess the association of risk factors (ERAS elements) with LOS.

A value of *p* < 0.05 was considered for significant differences.

## Results

### Demographics

A total of 192 patients were included, there were 96 patients in the ERAS group (45 men and 50 women, mean age 72.39 ± 6.12 years, mean BMI25.67 ± 3.32) and 96 patients in the non-ERAS group (40 men and 55 women, mean age 70.81 ± 6.27 years, mean BMI 25.73 ± 4.00). All surgeries were performed by a senior surgeon. Preoperative characteristics were similar between the two groups (Table 1). Demographic data were compared, and no statistically significant intergroup differences were observed. And there were no significant differences with comorbidities, ASA grade, or the number of fusion levels. The mean ERAS group and non-ERAS group operative time and intraoperative blood loss showed no significant difference. In addition, the mean preoperative JOA, VAS for the back and legs, and ODI score showed no significant difference (Table 1).

### Compliance to ERAS pathway

Our ERAS protocol included 12 elements interventions overall pathway compliance was 92.1% (Table 2). Patient education and counseling, no prolonged fasting, antimicrobial prophylaxis, and all intraoperative ERAS items were used in 100% of cases. The item with the lowest compliance was early removal of bladder catheter (52.6%).

**Table 2:** ERAS pathway compliance

Compliance with the ERAS program	
Variable	*n* (%)
Preoperative ERAS items	
Patient education and counseling	95 (100)
No prolonged fasting	95 (100)
Fluid and carbohydrate loading	90 (94.7)
Antithrombotic stockings	95 (100)
Antimicrobial prophylaxis	95 (100)
Intraoperative ERAS items	
Tranexamic acid	95 (100)
Maintenance of normothermia	95 (100)
Local infiltration analgesia	95 (100)
Postoperative ERAS items	
Early ambulation	63 (66.3)
Early removal of bladder catheter	50 (52.6)
Early oral feeding	70 (73.7)
Perioperative multimodal analgesia	93 (97.9)
Overall compliance (rate)	92.1

### Outcomes

The main clinical outcomes are shown in Table 3, after the implementation of ERAS, there was no significant difference in complication and mortality between ERAS group and non-ERAS group. Furthermore, the mean postoperative JOA, VAS for the back and legs, ODI, and readmission rates score showed no significant difference at 30-day follow-up, as complete data were available for 83% of patients at this early time point. However, we observed a statistically significant decrease in LOS in the ERAS group (12.30 ± 3.03 in ERAS group versus 15.50 ± 1.88 in non-ERAS group, *p* = 0). Multivariable linear regression showed that comorbidities (*p* = 0.023) and implementation of ERAS program (*p* = 0.002) were correlated with prolonged LOS. On the other hand, age (*p* = 0.379), sex (*p* = 0.085), BMI (*p* = 0.535), smoker (*p* = 0.137), ASA ≥ 3 (*p* = 0.062), fusion number (*p* = 0.236), operative time (*p* = 0.151), blood loss (*p* = 0.079), preoperative JOA (*p* = 0.235), preoperative VAS back (*p* = 0.949), preoperative VAS leg (*p* = 0.656), and preoperative ODI (*p* = 0.179) were not related to LOS. Multivariable logistic regression showed that no characteristics were associated with complications (Table 4).

**Table 3:** Postoperative outcomes

Outcome measure	ERAS	Non-ERAS	*p*
**Complications**			
Cerebrovascular accident	0	1	0.32
Cardiac arrest	0	0	
Deep vein thrombosis	0	1	0.32
Surgical site infection	1	4	0.17
Spinal fluid leakage	1	2	0.56
Neurological	1	1	1
LOS	12.30 ± 3.03	15.50 ± 1.88	0
30-day readmissions	1	1	
30-day mortality	0	0

**Table 4:** Multivariable analyses for LOS and complications

Characteristic	Multivariable linear regression for LOS		Multivariable logistic regression for any complications	
	Coefficient (95% CI)	*p* value	OR (95% CI)	*p* value
Age	0.24 (− 0.13 to 0.26)	0.379	1.15 (0.89–1.26)	0.563
Female	1.28 (− 0.51 to 1.10)	0.085	1.07 (0.91–1.14)	0.210
BMI	− 0.033 (− 0.13 to 0.07)	0.535	0.97 (0.92–1.06)	0.085
Smoker	0.76 (− 0.21 to 1.11)	0.137	2.10 (0.85–3.24)	0.121
Comorbidities	1.26 (0.29 to 2.23)	0.023	1.56 (0.77–2.91)	0.074
ASA ≥ 3	0.98 (− 0.03 to 1.92)	0.062	2.31 (0.98–4.53)	0.060
Fusion number	2.15 (− 1.29 to 3.37)	0.236	1.98 (0.91–2.58)	0.140
Operative time	0.36 (− 0.19 to 1.08)	0.151	1.33 (0.86–3.46)	0.088
Blood loss	1.12 (− 2.56 to 4.95)	0.079	1.44 (0.65–1.90)	0.872
ERAS	− 2.98 (− 3.76 to − 1.64)	0.002	0.72 (0.31–1.09)	0.077
Preoperative JOA	0.34 (− 0.46 to 0.88)	0.235	0.81 (0.70–1.34)	0.179
Preoperative VAS (back)	0.65 (− 0.70 to 2.01)	0.949	1.12 (0.81–2.03)	0.235
Preoperative VAS (leg)	0.98 (0.01 to 1.37)	0.656	2.09 (0.95–2.71)	0.068
Preoperative ODI (%)	− 0.02 (− 0.07 to 0.01)	0.179	1.36 (0.74–2.28)	0.307

## Discussion

A loss of disk height occurs with aging and may place non-physiological loads on adjacent segments as well as the facet joints, a common source of low back pain. Low back pain and sciatica can significantly impair psychosocial functioning, lead to sleep disorders and depressive symptoms, and may be linked to coronary heart disease, particularly in elderly persons [8]. However, due to poor physical function and comorbidities of the elderly persons, the lumbar spinal surgery in elderly patients has been associated with high rates of perioperative complications [9]. Worley et al. found that patients age > 65 had an increased risk of inpatient morbidity [10]. Initially proposed by Danish surgeon, Henrik Kehle, ERAS is a multi-professional and multidisciplinary approach to the care of the surgical patient in order to obtain a rapid recovery after surgical intervention [11]. While the initial efforts focused on colorectal surgery, the basic principles have now been adopted to multiple surgical specialties [6, 12–14]. ERAS protocols have been shown to be particularly beneficial for the elderly people who often have comorbidities and run a higher risk of surgical complications. Furthermore, these principles have been applied in patients with total hip arthroplasty, total knee arthroplasty, and those with intertrochanteric fracture, who not only experienced reduced hospital LOS, but also in improvement in patient care and reduced health care costs [15, 16].

At its core, the ERAS program aims at faster recovery, and LOS was used as the primary efficacy parameter. This study showed that application of ERAS in elderly patients with short-level lumbar fusion could decrease the LOS.

Shorting fasting and feeding time is one of important preoperative elements in our ERAS program. Because of perioperative starvation induces stress hormones release of the inflammatory cytokine and the accumulation of lipid products in skeletal muscles, traditional preoperative fasting for at least 8 h and oral feeding on postoperative 1 day may cause insulin resistance and metabolic stress [17, 18]. Insulin resistance and metabolic stress could increase the rate of postoperative complications [19]. Shortening preoperative fasting and postoperative eating time may shifts cellular metabolism to a more anabolic state [20]. Therefore, it can minimize protein loss, improve patient comfort, and decrease insulin resistance [21]. Good nutritional status could reduce the occurrence of complications such as wound infections and may help wound healing [22]. Research examining shortening preoperative fasting and postoperative eating time of elderly patients with lumbar surgery is markedly lacking, despite studies indicating that it is safe and effective [1, 18, 23]. Our studies showed that elderly patients with oral carbohydrate drink 2 h before the induction anesthesia and after surgery drinking water starting 2–4 h and early feeding started at 6 h is safe and without increasing complications.

Compared to other reports of ERAS in spine surgery, our ERAS program early mobilization compliance was high. Early mobilization is considered a key element of postoperative care in our ERAS program. Traditional long stay in bed was associated with infections and muscle weakness. Although a wealth of data confirms that early mobility could reduce the incidence of many of these complications and that early mobility within 24 h after spinal surgery is safe [24–26], there are few studies that investigate how early elderly patients can safely get out of bed and ambulate, and the way of the elderly patients get out of bed and ambulate. In this study, our early mobility protocol was from sitting out of bed or walking with assistance to walking without assistance within 24 h. Early mobilization following surgery has multiple benefits including improved ventilation, muscle strength, and functional capacity [24]; our results showed that early mobilization in elderly patients after short-level lumbar fusion is safe and without increasing complications and 30-day readmission rates.

However, this study has several limitations. This study is the retrospective design, small sample size. Given the lack of long-term follow-up data, definitive conclusions cannot be drawn beyond a 30-day period. Furthermore, the ERAS and non-ERAS groups were assessed at different times, which may have introduced analytical bias. Further multicenter studies with a larger participant population are required to confirm the safety and efficacy of our ERAS protocol in elderly patients after short-level lumbar fusion surgery.

## Conclusions

In conclusion, this report describes the first ERAS protocol used in elderly patients after short-level lumbar fusion surgery. Our ERAS program is safe and could help decreases LOS in elderly patients with short-level lumbar fusion. Further studies with more participants are required to validate these findings. While still in its infancy, with modified approaches to our ERAS protocol, will likely improve adherence to the protocol and outcomes.

## Data Availability

All data generated or analyzed during this study are included in this published article

## Abbreviations

ERAS: Enhanced recovery after surgery
LOS: Length of stay
BMI: Body mass index
ASA: American Society of Anesthesiologists
JOA: Japanese Orthopedic Association
ODI: Oswestry Disability Index
VAS: Visual analog score

## References

[1] Xu B, Xu WX, Lao YJ, Ding WG, Lu D, Sheng HF (2019). Multimodal nutritional management in primary lumbar spine surgery: a randomized controlled trial. Spine (Phila Pa 1976)..

[2] Adogwa O, Elsamadicy AA, Vuong VD, Moreno J, Cheng J, Karikari IO (2017). Geriatric comanagement reduces perioperative complications and shortens duration of hospital stay after lumbar spine surgery: a prospective single-institution experience. J Neurosurg Spine..

[3] Rihn JA, Hilibrand AS, Zhao W, Lurie JD, Vaccaro AR, Albert TJ (2015). Effectiveness of surgery for lumbar stenosis and degenerative spondylolisthesis in the octogenarian population. J Bone Joint Surg Am.

[4] Dietz N, Sharma M, Adams S, Alhourani A, Ugiliweneza B, Wang D (2019). Enhanced recovery after surgery (ERAS) for spine surgery: a systematic review. World Neurosurg..

[5] Gerhardt J, Bette S, Janssen I, Gempt J, Meyer B, Ryang YM (2018). Is eighty the new sixty? Outcomes and complications after lumbar decompression surgery in elderly patients over 80 years of age. World Neurosurg..

[6] Ljungqvist O, Hubner M (2018). Enhanced recovery after surgery-ERAS-principles, practice and feasibility in the elderly. Aging Clin Exp Res..

[7] Soffin EM, Vaishnav AS, Wetmore DS, Barber L, Hill P, Gang CH (2019). Design and implementation of an enhanced recovery after surgery (ERAS) program for minimally invasive lumbar decompression spine surgery: initial experience. Spine (Phila Pa 1976)..

[8] Cloyd JM, Acosta FL, Ames CP (2008). Complications and outcomes of lumbar spine surgery in elderly people: a review of the literature. J Am Geriatr Soc..

[9] Drazin D, Al-Khouja L, Lagman C, Ugiliweneza B, Shweikeh F, Johnson JP (2016). Scoliosis surgery in the elderly: complications, readmissions, reoperations and mortality. J Clin Neurosci..

[10] Worley N, Marascalchi B, Jalai CM, Yang S, Diebo B, Vira S (2016). Predictors of inpatient morbidity and mortality in adult spinal deformity surgery. Eur Spine J..

[11] Bardram L, Funch-Jensen P, Jensen P, Crawford ME, Kehlet H (1995). Recovery after laparoscopic colonic surgery with epidural analgesia, and early oral nutrition and mobilisation. Lancet..

[12] Keil DS, Schiff LD, Carey ET, Moulder JK, Goetzinger AM, Patidar SM (2019). Predictors of admission after the implementation of an enhanced recovery after surgery pathway for minimally invasive gynecologic surgery. Anesth Analg..

[13] Stein MJ, Frank SG, Lui A, Zhang T, Zhang J (2019). Ambulatory latissimus dorsi flap breast reconstruction: a prospective cohort study of an enhanced recovery after surgery (ERAS) protocol. J Plast Reconstr Aesthet Surg..

[14] Parise P, Cinelli L, Ferrari C, Cossu A, Puccetti F, Garutti L (2020). Early red flags associated with delayed discharge in patients undergoing gastrectomy: analysis of perioperative variables and ERAS protocol items. World J Surg..

[15] Kang Y, Liu J, Chen H, Ding W, Chen J, Zhao B (2019). Enhanced recovery after surgery (ERAS) in elective intertrochanteric fracture patients result in reduced length of hospital stay (LOS) without compromising functional outcome. J Orthop Surg Res..

[16] Vendittoli PA, Pellei K, Desmeules F, Masse V, Loubert C, Lavigne M (2019). Enhanced recovery short-stay hip and knee joint replacement program improves patients outcomes while reducing hospital costs. Orthop Traumatol Surg Res..

[17] Ljunggren S, Hahn RG (2014). Nystrom T:Insulin sensitivity and beta-cell function after carbohydrate oral loading in hip replacement surgery: a double-blind, randomised controlled clinical trial. Clin Nutr..

[18] Fujikuni N, Tanabe K, Tokumoto N, Suzuki T, Hattori M, Misumi T (2016). Enhanced recovery program is safe and improves postoperative insulin resistance in gastrectomy. World J Gastrointest Surg..

[19] Pillinger NL, Robson JL, Kam P (2018). Nutritional prehabilitation: physiological basis and clinical evidence. Anaesth Intensive Care..

[20] Scott MJ, Baldini G, Fearon KC, Feldheiser A, Feldman LS, Gan TJ (2015). Enhanced recovery after surgery (ERAS) for gastrointestinal surgery, part 1: pathophysiological considerations. Acta Anaesthesiol Scand..

[21] Alito MA, de Aguilar-Nascimento JE (2016). Multimodal perioperative care plus immunonutrition versus traditional care in total hip arthroplasty: a randomized pilot study. Nutr J..

[22] Bohl DD, Shen MR, Mayo BC, Massel DH, Long WW, Modi KD (2016). Malnutrition predicts infectious and wound complications following posterior lumbar spinal fusion. Spine (Phila Pa 1976)..

[23] Cao G, Huang Q, Xu B, Huang Z, Xie J, Pei F (2017). Multimodal nutritional management in primary total knee arthroplasty: a randomized controlled trial. J Arthroplasty..

[24] Ramos Dos Santos PM (2017). Aquaroni Ricci N, Aparecida Bordignon Suster E, de Moraes Paisani D, Dias Chiavegato L: Effects of early mobilisation in patients after cardiac surgery: a systematic review. Physiotherapy..

[25] Castelino T, Fiore JF, Niculiseanu P, Landry T, Augustin B, Feldman LS (2016). The effect of early mobilization protocols on postoperative outcomes following abdominal and thoracic surgery: a systematic review. Surgery..

[26] Rupich K, Missimer E, O'Brien D, Shafer G, Wilensky EM, Pierce JT (2018). The benefits of implementing an early mobility protocol in postoperative neurosurgical spine patients. Am J Nurs..

